# A temporal network analysis of complex post-traumatic stress disorder and psychosis symptoms

**DOI:** 10.1017/S0033291725000030

**Published:** 2025-02-20

**Authors:** Peter Panayi, Alba Contreras, Emmanuelle Peters, Richard Bentall, Amy Hardy, Katherine Berry, William Sellwood, Robert Dudley, Eleanor Longden, Raphael Underwood, Craig Steel, Hassan Jafari, Liam Mason, Filippo Varese

**Affiliations:** 1 Division of Psychology and Mental Health, Manchester Academic Health Sciences Centre, University of Manchester, Manchester, UK; 2 Complex Trauma and Resilience Research Unit, Greater Manchester Mental Health NHS Foundation Trust, Manchester, UK; 3 Department of Psychobiology and Methodology of Behavioural Sciences, University of Malaga, Malaga, Spain; 4 Department of Psychology, King’s College London, London, UK; 5 South London and Maudsley NHS Foundation Trust, London, UK; 6 Department of Psychology, University of Sheffield, Sheffield, UK; 7 Division of Health Research,Faculty of Health & Medicine, University of Lancaster Lancaster, UK; 8 Department of Psychology, University of York, York, UK; 9 Oxford Centre for Psychological Health, Oxford Health NHS Foundation Trust, Oxford, UK; 10 Oxford Institute of Clinical Psychology Training and Research, University of Oxford, Oxford, UK; 11 Department of Biostatistics and Health Informatics, Institute of Psychiatry, Psychology and Neuroscience, King’s College London, London, UK; 12 Division of Psychology & Language Sciences, University College London, London, UK

**Keywords:** disturbances of self-organization, experience sampling methodology, hallucinations, paranoia, trauma, visions

## Abstract

**Background:**

Symptoms of complex post-traumatic stress disorder (cPTSD) may play a role in the maintenance of psychotic symptoms. Network analyses have shown interrelationships between post-traumatic sequelae and psychosis, but the temporal dynamics of these relationships in people with psychosis and a history of trauma remain unclear. We aimed to explore, using network analysis, the temporal order of relationships between symptoms of cPTSD (i.e. core PTSD and disturbances of self-organization [DSOs]) and psychosis in the flow of daily life.

**Methods:**

Participants with psychosis and comorbid PTSD (*N* = 153) completed an experience-sampling study involving multiple daily assessments of psychosis (paranoia, voices, and visions), core PTSD (trauma-related intrusions, avoidance, hyperarousal), and DSOs (emotional dysregulation, interpersonal difficulties, negative self-concept) over six consecutive days. Multilevel vector autoregressive modeling was used to estimate three complementary networks representing different timescales.

**Results:**

Our between-subjects network suggested that, on average over the testing period, most cPTSD symptoms related to at least one positive psychotic symptom. Many average relationships persist in the contemporaneous network, indicating symptoms of cPTSD and psychosis co-occur, especially paranoia with hyperarousal and negative self-concept. The temporal network suggested that paranoia reciprocally predicted, and was predicted by, hyperarousal, negative self-concept, and emotional dysregulation from moment to moment. cPTSD did not directly relate to voices in the temporal network.

**Conclusions:**

cPTSD and positive psychosis symptoms mutually maintain each other in trauma-exposed people with psychosis via the maintenance of current threat, consistent with cognitive models of PTSD. Current threat, therefore, represents a valuable treatment target in phased-based trauma-focused psychosis interventions.

## Introduction

Traumatic life experiences are highly common among people with psychosis, with dose–response relationships between traumatic life events and psychosis risk indicating a potential causal link (Pastore, de Girolamo, Tafuri, Tomasicchio, & Margari, 2022). Accordingly, systematic reviews indicate that symptoms of post-traumatic stress disorder (PTSD) play a role in the pathway from trauma to psychosis (Alameda et al., 2020; Bloomfield et al., 2021; Sideli et al., 2020). This may explain the phenomenological overlap between psychotic symptoms and post-traumatic sequelae, such as voices that overlap literally or thematically with traumatic memories; or a shared sense of current threat characterizing both paranoia and post-traumatic hypervigilance (Compean & Hamner, 2019; Van Den Berg et al., 2023). Psychological interventions for psychosis are becoming increasingly trauma-focused to adequately address this overlap and improve outcomes (Hardy et al., 2023).

In the latest International Classification of Disease (11th Edition; ICD-11), so-called ‘core’ symptoms of PTSD include re-experiencing of traumatic events (e.g. memory intrusions, flashbacks), avoidance of reminders of the events (either internally or externally), and hyperarousal (e.g. hypervigilance) (World Health Organization [WHO], 2019). The trauma histories typical of people with psychosis (i.e. repeated interpersonal victimization often during childhood) pose an increased risk of complex PTSD (cPTSD), classified in the latest ICD (Karatzias et al., 2019; Trauelsen et al., 2015). cPTSD includes the aforementioned ‘core’ symptoms of PTSD, alongside disturbances of self-organization (DSOs): emotional dysregulation, negative self-concept, and interpersonal difficulties (WHO, 2019). Emerging evidence suggests cPTSD may be more common among people with psychosis than PTSD, and that DSOs play a role in the trauma–psychosis pathway (Panayi et al., 2022). Like core PTSD symptoms, there is phenomenological overlap between psychotic symptoms and DSOs (e.g. affective blunting in negative symptoms of psychosis and emotional dysregulation in DSOs) (Favrod et al., 2019).

To theoretically capture this overlap and guide intervention, psychological models of PTSD in psychosis have been developed. In a seminal model, Morrison and colleagues proposed that traumatic memory intrusions may be appraised as a threatening sensory experience occurring in the present, leading to distressing or unhelpful coping strategies that maintain the experience (Morrison, Frame, & Larkin, 2003). This model has since been expanded following developments in the field that indicate multiple trauma-related mechanisms may maintain psychosis, including DSO-consistent factors (e.g. emotional dysregulation, negative self-concept) that may perpetuate intrusive trauma memories, their appraisals, and subsequent coping strategies (Hardy, 2017). Recent analytic innovations may be applied to explore complex interrelationships between symptoms of cPTSD and psychosis, and test whether they mirror relationships proposed by theoretical models to guide promising new directions for trauma-focused psychosis interventions.

Network theory, a relatively new perspective in mental health research, proposes that mental health difficulties arise from self-perpetuating relationships between individual symptoms as opposed to an underlying ‘disease’ entity (Borsboom, 2017). These relationships between elements can be represented in a network of variables, where symptoms are represented by circles (i.e. ‘nodes’) connected by lines (i.e. ‘edges’) that demonstrate the strength and direction of their relationship. Derived from network theory, network analysis is a data-driven statistical technique that can be used to visualize such networks, identifying the importance of each node in maintaining the network (Fried & Cramer, 2017). Further metrics can be derived to gain further insight on the structure of the network, such as clustering (identification of subcommunities of closely related nodes based on relative edge strength; Golino & Epskamp, 2017) and centrality (quantifying the relative importance of each node in the network; Bringmann et al., 2016).

Network analysis has been increasingly used to model the complexity of psychological difficulties, with metrics such as clustering applied to identifying psychological symptom communities/domains, and centrality to the identification of potentially tractable targets for mental health interventions (Hardy, O’Driscoll, Steel, Van Der Gaag, & Van Den Berg, 2021; Levin et al., 2021; McElroy et al., 2019). Several studies have already applied network analysis to examine the interrelationships between PTSD and psychosis in cross-sectional samples. Consistent with the models outlined above, psychotic and PTSD symptoms are often bridged by post-traumatic hyperarousal and trauma-related beliefs (Astill Wright et al., 2023; Hardy et al., 2021; Jin et al., 2022). Hyperarousal and negative trauma-related beliefs are arguably consistent with DSOs (i.e. emotional dysregulation and negative self-concept, respectively), tentatively modeling the overlap between cPTSD and psychosis. A recent network analysis explicitly modeled this overlap using the only validated diagnostic measure of cPTSD in a trauma-exposed sample of people with comorbid psychosis and PTSD (Frost, O’Driscoll, Peters, & Hardy, 2023). Affective dysregulation was related to voices, and negative self-concept linked to delusions *via* self-blame, affirming the importance of trauma-related mechanisms beyond episodic memory (i.e. DSOs and trauma-related beliefs) in the above models of post-traumatic stress in psychosis.

Like most network analyses, networks of (c)PTSD and psychosis to date have been cross-sectional (Contreras, Nieto, Valiente, Espinosa, & Vazquez, 2019; Frost et al., 2023; Hardy et al., 2021). However, the lack of temporality in cross-sectional networks limits insight into the directionality of psychological mechanisms that could clarify processes of symptom formation and maintenance. A growing number of studies therefore use longitudinal data to capture the dynamic interplay between symptoms using temporal network models (Blanchard, Contreras, Kalkan, & Heeren, 2022; Epskamp, van Borkulo, et al., 2018). In contrast to traditional longitudinal designs capturing developmental change over extended periods, intense longitudinal designs (i.e. multiple measurements recorded over short periods) better suit the self-perpetuating stability of psychopathological networks (Epskamp, 2020). Experience sampling methodology (ESM) is well-placed to capture data of this kind, involving several measurements per day over a given period (Myin-Germeys et al., 2018; Trull & Ebner-Priemer, 2009). Several studies have applied temporal network analysis to ESM data using multilevel vector autoregressive (mlVAR) modeling to model the dynamic relationships between individual symptoms in the flow of daily life (Blanchard et al., 2022).

The application of temporal network analysis to intensive longitudinal data generates three complementary networks: between-subject, contemporaneous, and temporal networks (Epskamp, Borsboom, & Fried, 2018). Between-subjects networks depict partial correlations between symptoms on average throughout the testing period, and as such can be used to visualize relationships that may manifest over longer periods (Epskamp & Fried, 2018; Epskamp, Waldorp, Mõttus, & Borsboom, 2018). On the other hand, contemporaneous networks depict partial correlations between symptoms within a single moment, and temporal networks depict regression coefficients of relationships between symptoms from one moment to the next (i.e. ‘lagged’ relationships) (Epskamp & Fried, 2018; Epskamp, Waldorp, et al., 2018). Relationships between symptoms may operate over small timeframes, and as such emerge in contemporaneous, but not temporal, networks (Epskamp, van Borkulo, et al., 2018). Conversely, relationships may also operate over longer periods and as such emerge in the between-subjects network, but not in contemporaneous or temporal networks.

The aforementioned cross-sectional network analysis of cPTSD and psychotic symptoms analyzed baseline clinical data from a randomized controlled trial of trauma-focused cognitive-behavioral therapy for psychosis (Frost et al., 2023; Peters et al., 2022). A subsample of this group completed an additional ESM study prior to randomization, which found that increases in both core PTSD and DSOs temporally predicted subsequent increases in paranoia, voices, and visions in the flow of daily life (Panayi et al., 2024). Yet, several queries remain about the dynamics of cPTSD and psychotic symptoms, including the interdependence of effects (given models were tested separately for each positive symptom), symptom-specific effects (since core PTSD and DSOs were entered as two composite scores), and directionality of effects (since models were tested unidirectionally). By considering each symptom as an individual node, testing relationships between symptoms controlling for others in the network, and estimating networks over different timeframes, temporal network analysis is well-placed to address these queries and comprehensively assess cPTSD–psychosis symptom dynamics.

In summary, there have been no attempts to scrutinize the interrelations between post-traumatic sequelae and psychotic symptoms using temporal networks, and networks of this kind could clarify uncertainties around the potentially maintaining effect of cPTSD in trauma-exposed people with psychosis. This study therefore applied temporal network analysis to an ESM dataset collected via a clinical trial to establish the temporal dynamics of relationships between symptoms of cPTSD and psychosis. Specifically, we aimed to determine the persistence of relationships across different networks reflecting disparate timeframes as well as the potential temporal order of relationships between symptoms.

## Method

### Design

An intense-longitudinal ESM design was used (Myin-Germeys et al., 2018), wherein repeated measures of positive symptoms of psychosis (i.e. paranoia, voices, and visions), core PTSD (intrusive memories, avoidance, hyperarousal), and DSOs (emotional dysregulation, negative self-concept, and interpersonal difficulties) were taken up to 10 times a day over six consecutive days using a mobile app. A full description of the ESM protocol is described in Panayi et al. (2024).

### Participants

Data for this study were collected *via* the Study of Trauma and Recovery (STAR) trial, a randomized controlled trial testing the efficacy of and mechanisms underlying trauma-focused cognitive-behavioral therapy for psychosis (Peters et al., 2022). STAR trial participants were mental health service users recruited from five sites across the United Kingdom. A subsample of 153 participants who consented to additional ESM procedures prior to randomization were included in this study. Demographic and clinical characteristics are listed in Table 1; comparisons with the wider STAR sample are described in Panayi et al. (2024).

**Table 1.** Demographic and clinical characteristics (*N* = 153)

Demographic		%
Gender	Male	38.56
	Female	56.86
	Nonbinary	2.61
	Prefer not to say	1.96
Ethnicity	White British	73.20
	Black (African or Caribbean)	9.15
	Mixed heritage	4.58
	South Asian (Indian or Pakistani)	2.61
	Other	9.15
	Prefer not to say	1.31
Relationship status	Single	57.52
	Cohabiting/Married/Civil partnership	30.07
	Separated/Divorced/Widowed	11.11
	Prefer not to say	1.31
Education	Primary education	2.61
	Secondary education	30.07
	Vocational education/college	39.22
	Higher education	23.53
	Other	2.61
	Prefer not to say	1.96
Employment status	Working	18.30
	Studying	5.23
	Volunteering	4.58
	Caregiver	-
	Retired	1.96
	Not currently working	69.28
	Prefer not to say	0.65
Schizophrenia spectrum diagnosis (F20–29; ICD–10)	Schizophrenia (F20)	15.13
	Persistent delusional disorder (F22)	1.32
	Schizoaffective disorder (F25)	9.87
	Other nonorganic psychotic disorder (F28)	30.92
	Unspecified nonorganic psychosis (F29)	43.42
Hears voices^a^		81.70
Sees visions^a^		63.40
Antipsychotic prescription		83.66
ITQ diagnosis	None	12.42
	PTSD	8.50
	cPTSD	79.08
Other diagnoses	Anxiety disorders (Generalized anxiety, OCD, Other anxiety disorders)	15.69
	Autism	7.84
	Bipolar	6.54
	Depression (with or without psychotic features)	37.91
	Personality disorders (including emotionally unstable and other personality disorders)	30.07
	Substance-related disorders	6.54
	Other (e.g. ADHD, eating disorders, severe stress and adjustment disorder)	10.46
Multiple trauma exposure	Repeated events (at least 1 TALE item endorsed ‘more than once’)	100
	Multiple trauma types	100
Trauma timing	Child (endorsed any TALE item <16)	92.81
	Adult (endorsed any TALE item 16 or over)	95.42
	Both (endorsed any TALE item <16 AND 16 or over)	88.24
		*M(SD)*
Age		37.00 (12.14)
Number of trauma types^b^		11.52 (3.20)
ITQ-PTSD		17.93 (4.16)
ITQ-DSO		18.63 (4.52)
GPTS-persecution		23.21 (11.40)
PSYRATS-visions		30.84 (5.89)
PSYRATS-voices		30.87 (5.90)

a Based on baseline PSYRATS data

b Based on baseline TALE data

*Note*: M, mean; SD, standard deviation; PTSD, post-traumatic stress disorder; DSOs, disturbances of self-organization; ITQ, International Trauma Questionnaire Cloitre et al. (2018); GPTS, Green et al. Paranoid Thoughts Scale Freeman et al. (2021); PSYRATS, Psychotic Symptom Rating Scales Haddock et al.(1999); TALE, Trauma and Life Events scale Carr et al. (2018).

### Inclusion criteria

All STAR trial participants met ICD-10 criteria for schizophrenia-spectrum diagnoses (F20–29) ascertained from the ICD-10 checklist by the research team, following clinical notes review and consultation with the care team, as appropriate, and scored ≥2 (‘moderate’ intensity) on the distress item of at least one psychotic symptom on the Psychotic Symptom Rating Scales (Haddock, McCarron, Tarrier, & Faragher, 1999). Participants also endorsed at least one traumatic life event on the Trauma And Life Events checklist (Carr, Hardy, & Fornells-Ambrojo, 2018) and met criteria for PTSD on the Clinician-Administered PTSD Scale for DSM-5 (Weathers et al., 2018). Participants were not eligible for the STAR trial if their psychotic or PTSD symptoms were primarily organic in etiology, had a primary substance misuse diagnosis, required an interpreter to engage with the trial, or (within the prior 3 months) had a major medication change or received trauma-focused therapies. There were no additional criteria for taking part in the additional ESM study other than provision of informed consent for this additional ESM study. Of note, 31% of participants were recruited from Early Intervention for Psychosis (EIP) services in England, where best practice guidance advises against the use of potentially stigmatizing labels (e.g. schizophrenia, schizoaffective disorder) among those experiencing first-episode psychosis. Instead, ICD-10 F28 (other nonorganic psychotic disorder) or F29 (unspecified psychotic disorder) categories are routinely applied.

### Baseline clinical measures

Specific baseline measures administered as part of the STAR trial assessment battery were used here for descriptive purposes. These include the Trauma and Life Events Checklist (Carr et al., 2018) to assess the occurrence of difficult life experiences, the International Trauma Questionnaire (Cloitre et al., 2018) to assess the presence and severity of PTSD and DSOs, Green et al. Paranoid Thoughts Scale-Revised (Freeman et al., 2021) to measure paranoia and Psychotic Symptom Rating Scales (PSYRATS; Haddock et al., 1999) to measure voice hearing. The STAR trial added an adapted PSYRATs version capturing the presence of hallucinations in other modalities, used here to measure vision seeing (Tsang, n.d.). A full description of the measures used in the STAR assessment battery is described in Peters et al. (2022) and their application to the current study in Panayi et al. (2024).

### ESM measures

The ESM questionnaire involved 29 questions assessing nine domains; items used for this study are documented in Table 2. All items used in the analyses were scored on a 7-point Likert scale from 1 (‘Not at all’) to 7 (‘Very much so’). Items were based on previous studies of similar populations (Chun, 2016; Kimhy et al., 2006), amended in consultation with stakeholders, including people with lived experience of trauma and psychosis, clinicians, and researchers. For an explanation of item derivation and validation, see Panayi et al. (2024).

**Table 2.** Breakdown of variables analyzed in this study and corresponding ESM items

Construct	ESM item(s)
*Psychosis*	
Paranoia	*Right now I feel suspicious* *Right now I believe that some people want to hurt me deliberately*
Hallucinations	*Right now I can hear a voice or voices that other people cannot hear* *Right now I see things that other people cannot see*
*PTSD*	
Intrusive memories	*Since the last beep, unwanted memories about the experience popped into my mind*
Avoidance	*Since the last beep, I avoided thoughts, feelings, or physical sensations that remind me of the experience* *Since the last beep, I avoided places, people, or situations that remind me of the experience*
Hyperarousal	*Since the last beep, I felt super-alert, watchful, or on guard* *Since the last beep, I felt jumpy or easily startled*
*DSO*	
Emotional dysregulation	*Since the last beep I found it difficult to control my emotions* *Since the last beep I felt spaced out, numb, or emotionally shut down*
Interpersonal difficulties	*Since the last beep I found it easy to stay emotionally close to people^a^* *Since the last beep I felt distant or cut off from people*
Negative self-concept	*Right now I feel ashamed* *Right now I believe I am a good person^a^*

a Reverse scored.

*Note:* PTSD, Post-traumatic stress disorder; DSOs, disturbances of self-organization.

### Procedure

Participants were recruited from five National Health Service (NHS) Mental Health Trusts across the UK (NHS Research Ethics Committee ref: 20/LO/0853). The study involved an intensive longitudinal, time-contingent ESM design, consisting of up to 10 observations per day over a 6-day period. Observations were scheduled in a quasi-random nature, delivered randomly within consecutive 90-minute blocks adjusted to suit the waking hours of individual participants, in accordance with ESM best practice (Dejonckheere & Erbas, 2022).

After providing informed consent for the ESM study, participants were supported by a researcher to download a mobile app, m-Path (Mestdagh et al., 2023), onto their personal mobile phone or were provided a study phone. After completing a practice questionnaire to clarify understanding of the questions and resolve any queries, the researcher scheduled the notifications during the participants’ typical waking hours. Participants were then invited to complete ESM questionnaires administered via the app for six consecutive days, starting the following day. To address potential technical issues or promote procedural compliance, participants received a phone call on the second day of the study, and the response rate was monitored throughout the 6-day data collection period. All participants were debriefed and reimbursed at the end of the study.

### Data analysis

The analyses were conducted in *R* 4.2.1 (R Core Team, 2023); the R code is presented in Supplementary Material 1. The *mlVAR* package (version 0.5.1; Epskamp, Deserno, et al., 2023) was used to estimate the temporal network models of cPTSD and psychosis symptoms. Shapiro–Wilk tests were used to assess normality in each variable. The assumption of stationarity (i.e. that variable means and standard deviations remain constant over time; Aalbers et al., 2019; Bringmann et al., 2016) was tested via Kwiatkowski–Phillips–Schmidt–Shin (KPSS) unit root tests using the *R* package *tseries* (version 0.54; Trapletti & Hornik, 2023).

To establish the temporal dynamics of cPTSD and psychotic symptoms, *mlVAR* was used to generate three complementary networks reflecting three timeframes: (1) a between-subjects network, i.e. Gaussian Graphical Model (GGM) composed of regularized partial correlations (after taking into account the remaining variables in the network) between participants’ means during the 6-day study duration period; (2) a contemporaneous network based on a GGM comprising edges that depict multilevel partial correlations between nodes in a single ESM measurement point (referred to as ‘moments’), after controlling for other contemporaneous and temporal associations; (3) a temporal network based on regression coefficients of lagged relationships between nodes from one timepoint to the next after controlling for all other nodes at the previous timepoint (Epskamp, Borsboom, et al., 2018; Epskamp, Waldorp, et al., 2018).

Model coefficients were plotted as graphical networks by *mlVAR* using the *qgraph* package (version 1.9.5; Epskamp, Costantini, et al., 2023). Contemporaneous and between-subjects models were estimated conservatively, using the ‘AND-rule’ approach, which retains edges if both regressions on which the edge is based are significant (*α* = .05). Clustering metrics were applied to the between-subjects network using exploratory graph analysis (EGA) via the *EGAnet* package (Golino & Christensen, 2022) to avoid temporal interference while maintaining fullness of the data.

## Results

### Descriptive statistics

Means and standard deviations of within-participant means and standard deviations for each variable are presented in Table 3.

**Table 3.** Means and standard deviations of within-participant means and within-participant standard deviations for all ESM variables

Variable		*M (SD)*	*SD (SD)*
PTSD	Memory intrusions	4.16 (1.43)	1.46 (0.65)
	Avoidance	4.29 (1.50)	1.09 (0.54)
	Hyperarousal	4.12 (1.41)	1.08 (0.57)
DSO	Emotional dysregulation	4.13 (1.32)	1.05 (0.47)
	Interpersonal difficulties	3.96 (0.86)	0.82 (0.36)
	Negative self-concept	3.79 (1.31)	0.86 (0.42)
Psychosis	Paranoia	4.26 (1.45)	0.92 (0.50)
	Voices	3.87 (2.12)	0.95 (0.68)
	Visions	3.11 (1.98)	0.92 (0.67)

*Note:* M, mean; SD, standard deviation; PTSD, post-traumatic stress disorder; DSOs, disturbances of self-organization. All variables were measured using a 7-point Likert scale (range 1–7).

mlVAR estimation typically requires a minimum of 20 data points per participant, to prevent bias in within-person centering (Jordan, Winer, & Salem, 2020). Sensitivity analyses were conducted excluding participants with fewer than 20 observations (*n* = 32), but this did not affect the outcomes of the analyses. As such, the following results are presented from the full dataset (*N* = 153). Other indicators of adherence to the ESM protocol are listed in Panayi et al. (2024).

### Assumptions

Shapiro–Wilk tests indicated that memory intrusions, hyperarousal, emotional dysregulation, and negative self-concept were normally distributed, whereas avoidance, interpersonal difficulties, voices, visions, and paranoia were not (see Supplementary Table 1 for normality values and Supplementary Figures 1 and 2 for histograms). The size of partial correlation coefficients suggested no variables were multicollinear in any network (Epskamp & Fried, 2018). KPSS tests suggested the data were stationary for all variables, except the DSO-emotional dysregulation negative self-concept subscales (see Supplementary Table 2 for KPSS test values). Multilevel regressions indicated extremely small increases (both *b*’s < .01) in these subscale scores as the measurement week progressed. Considering these effect sizes in combination with the KPSS probability values approaching nonsignificance (all *p*’s = .04) and the high Type I error of KPSS tests (Jordan et al., 2020), supplementary augmented Dickey–Fuller (ADF) tests were used to confirm these results, in line with best statistical practice for temporal network analysis (Blanchard et al., 2022). ADF tests confirmed trend and level stationarity of these subscales in all participants (all *p*’s < .01). Prior research suggests temporal networks may be robust against violations of these assumptions, but this remains unclear (Aalbers et al., 2019; Blanchard et al., 2022; Faelens, de Putte, Hoorelbeke, de Raedt, & Koster, 2021). These assumptions are therefore reported here in the interest of fullness and supporting efforts to establish the robustness of temporal networks.

## Network estimation and visualization

### Between-subjects network

**Figure 1.**
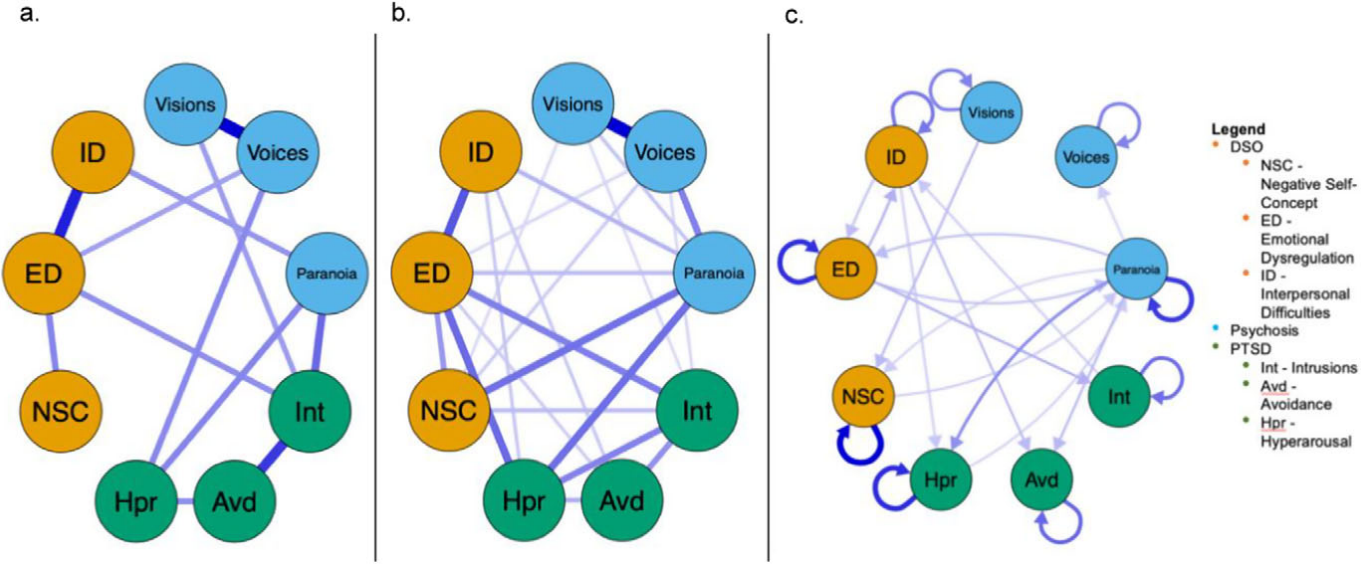
Graphical models representing (a) between-subjects network depicting average symptom relationships over the course of the testing period; (b) contemporaneous network depicting symptom relationships within a single moment, controlling for all other relationships in the network; (c) temporal network depicting symptom relationships between each moment, controlling for all relationships at the previous moment. Blue edges denote positive relationships; thicker edges denote stronger relationships; arrows denote the direction of prediction. Node colors represent symptom groups, not outcomes of clustering analysis.

The between-subjects network is illustrated in Figure 1a, depicting intraindividual correlations between mean levels of each node across the ESM testing period. This network suggests, on average throughout the week, paranoia correlated positively with intrusive memories and hyperarousal, whereas visions associated with the former and voices the latter. Regarding DSOs, the network indicates that paranoia correlated positively with interpersonal difficulties, and voices with emotional dysregulation, on average throughout the week. Network coefficients are presented in Supplementary Table 3.

### Contemporaneous network

The contemporaneous network – i.e. node associations from a single moment after controlling for other contemporaneous and temporal associations – is illustrated in Figure 1b. In this timeframe, relationships persisted for paranoia with hyperarousal and interpersonal difficulties, and (weakly) between visions and intrusive memories, and voices and emotional dysregulation. Voices, visions, and (most strongly) paranoia all newly related to negative self-concept in this network. New relationships also emerged between paranoia and emotional dysregulation, and between voices and intrusive memories. Network coefficients are presented in Supplementary Table 4.

### Temporal network

The temporal network – i.e. the predictive strength of each node from one moment (*t*) to the next (*t + 1*) – is illustrated in Figure 1c. Autoregressive loops depict the degree to which nodes predicted themselves in the interval between measurements; particularly strong loops for emotional dysregulation, negative self-concept, hyperarousal, and paranoia suggest these experiences may be self-perpetuating and as such remain relatively stable over time. Paranoia showed positive, reciprocal momentary relationships with emotional dysregulation, negative self-concept, and (most strongly) hyperarousal. Neither voices nor visions demonstrated reciprocal temporal relationships with other nodes in the network; visions solely predicted negative self-concept while voices were solely predicted by paranoia. Network coefficients are presented in Supplementary Table 5.

### Centrality metrics

Measures of centrality (i.e. the degree to which nodes predict or are predicted by other nodes in the network) were derived from the temporal network matrix. All nodes exerted some influence on the network, except voices and avoidance. Similarly, all nodes received some influence from the network, except visions. Paranoia demonstrated the highest in- and outstrength centrality in the network. Emotional dysregulation demonstrated the second-highest outstrength, while hyperarousal showed the second-highest in-strength. Centrality plots are provided in Supplementary Figure 3.

## Discussion

In a large ESM sample of 153 participants with comorbid PTSD and psychosis, we applied temporal network analysis to establish the temporal dynamics of relationships between symptoms of cPTSD and psychosis in the flow of daily life. Our between-subjects network suggested that, on average over the testing period, all cPTSD symptoms (aside from avoidance and negative self-concept) related to at least one positive psychotic symptom. Many average relationships persist at the momentary level, indicating symptoms of cPTSD and psychosis may co-occur, especially paranoia with hyperarousal and negative self-concept where associations are strongest. Associations between negative self-concept and paranoia, voices, and visions; between paranoia and emotional dysregulation; and between voices and intrusive memories were absent in the between-subjects network, suggesting these relationships may only manifest over shorter timeframes. The temporal network suggested that paranoia reciprocally predicted, and was predicted by, hyperarousal, negative self-concept and emotional dysregulation from moment to moment, as opposed to temporally unidirectional relationships. In contrast, cPTSD symptoms did not relate to voices in the temporal network, indicating these may co-occur, but not temporally predict one another. Likewise, visions were associated with intrusive memories in the between subjects, but not temporal, network, suggesting a temporal relationship may manifest over longer timeframes.

There is a consistent, strong relationship between paranoia and hyperarousal in all three networks, suggesting these experiences are closely interlinked in our sample. Paranoia and hypervigilance often co-occur, as individuals are likely to be on edge or on guard under interpersonal threat (Alsawy, Wood, Taylor, & Morrison, 2015; Freeman et al., 2013). Though, safety behaviors in response to threat, including hypervigilance, have been shown to counterintuitively maintain symptoms of both PTSD and psychosis (Beierl, Böllinghaus, Clark, Glucksman, & Ehlers, 2020; Greenburgh et al., 2021). Consistently, a prior cross-sectional network analysis demonstrated the bridging role of hyperarousal between PTSD and psychosis (Hardy et al., 2021). Our findings demonstrate temporality of these effects, indicating a reciprocal relationship between hyperarousal and paranoia in people with co-occurring cPTSD and psychosis symptoms that – according to centrality indices – may maintain the comorbidity. The maintaining role of paranoia/hyperarousal in this comorbidity somewhat contrasts with historic proposals that post-traumatic sequelae and psychosis overlap *via* intrusive trauma memories (Morrison et al., 2003). That said, our findings provide temporal evidence for the stipulated emphasis on current threat in cognitive models of PTSD and psychotic symptoms (Ehlers & Clark, 2000; Freeman, 2007; Hardy, 2017). Notably, positive psychotic symptoms insufficient to warrant a schizophrenia-spectrum diagnosis have been repeatedly documented among people with PTSD (Braakman, Kortmann, & Van Den Brink, 2009; Shevlin, Armour, Murphy, Houston, & Adamson, 2011). Whether our findings apply to models of psychosis in PTSD requires investigation, to indicate the need for adaptation of trauma interventions to target these experiences.

All three psychosis symptoms related to negative self-concept in the contemporaneous network. Prior networks demonstrated the bridging role of trauma-related beliefs (including negative self-concept) between PTSD and psychosis (Frost et al., 2023; Hardy et al., 2021). We extend these findings by demonstrating temporal dynamics of this relationship; namely, that psychological mechanisms at play between trauma-related beliefs and psychosis may operate quickly, hence their presence only in the contemporaneous network. The temporal network suggests that, over time, negative self-concept may impact voices indirectly *via* paranoia. Consistently, prior cross-sectional analyses demonstrate delusional beliefs effectively mediate the relationship between PTSD and hallucinations (Frost et al., 2023; Hardy et al., 2021) and with a previous ESM study that found that increases in delusional thinking preceded both visual and auditory hallucinations (Oorschot et al., 2012). For some, paranoia may be an expression of negative self-beliefs that prevents disconfirmation of (and thus maintains) said beliefs (Bentall, Corcoran, Howard, Blackwood, & Kinderman, 2001; Kesting, Bredenpohl, Klenke, Westermann, & Lincoln, 2013). The reciprocal relationship observed in the temporal network provides temporal evidence of this mutually maintaining effect in people with comorbid cPTSD and psychosis.

In contrast, the temporal network suggests that visions may unidirectionally precede negative self-concept from moment to moment. The potentially activating effect of visions on negative trauma-related beliefs about the self is uncontroversial given their thematic (if not direct) trauma-relatedness (Van Den Berg et al., 2023). The findings of the temporal network therefore offer support to modern conceptualizations of visions as disintegrated trauma memories in trauma-exposed people with psychosis (Hardy, 2017; Morrison et al., 2003). Accordingly, visions and memory intrusions did (at least weakly) co-occur in the contemporaneous network, with potential temporal relationships manifesting over longer periods, as these nodes were related in the between-subjects, but not temporal, network.

In addition to negative self-concept, our temporal network also implicates a temporal relationship between paranoia and emotional dysregulation. ESM studies have previously uncovered predictive effects of emotional dysregulation on paranoia (Kimhy et al., 2020; Panayi et al., 2024); our network suggests this is reciprocal. As above, this demonstrates the dynamic nature of cPTSD–psychosis symptom relationships: unhelpful emotion regulation strategies in response to paranoid ideation may paradoxically maintain distressing beliefs, and vice versa (Lim, Gleeson, Alvarez-Jimenez, & Penn, 2018). A cross-sectional network of cPTSD and psychosis in this sample did not show a direct relationship between emotional dysregulation and delusions, potentially indicating our findings are specific to paranoia, or the timeframes captured by temporal networks (Frost et al., 2023). That said, a prior temporal network analysis did not demonstrate momentary relationships of paranoia with emotional dysregulation or self-esteem (closely related to negative self-concept) (Contreras, Valiente, Heeren, & Bentall, 2020). This disparity is likely due to the subclinical nature of Contreras and colleagues’ sample, where additional protective factors may be present that prevent the development of clinically significant difficulties (perhaps mechanistically via the prevention of interrelationships between DSOs and paranoia, in light of our findings).

Like the aforementioned relationship between visions and intrusive memories, voices related to emotional dysregulation in the between-subjects and contemporaneous, but not temporal, network. Their co-occurrence aside, temporal relationships between these nodes may therefore manifest over longer periods, consistent with their relationship in meta-analyses and cross-sectional networks (Bloomfield et al., 2021; Frost et al., 2023). Emotion regulation moderates stress reactivity, especially among people with PTSD (Badour & Feldner, 2013; Shapero, Abramson, & Alloy, 2016). As such, the relationship between emotional dysregulation and voices in this study is consistent with the affective pathway to psychosis that suggests stress reactivity precipitates psychotic symptoms (Collip et al., 2013; Myin-Germeys & van Os, 2007). Our findings indicate this pathway may be especially reflective of people with comorbid cPTSD and psychosis.

An important consideration contextualizing the findings is the sample. The use of a clinical sample is a strength, given the relative centrality of paranoia emergent here that was not evident in prior network analyses of PTSD and psychosis in the general population (Astill Wright et al., 2023; Jin et al., 2022). That said, the clinical trial through which the data for this study were collected may have introduced sample bias, since inclusion in the trial (principally) required participants to meet criteria for DSM-5 PTSD alongside meeting diagnostic criteria for an ICD-10 schizophrenia spectrum disorder and current, distressing psychotic symptoms (Peters et al., 2022). The sample may therefore be particularly affected by their traumatic life experiences in ways that are not representative of all people experiencing psychosis. Indeed, in contrast to the present study, paranoia was not highly central in a cross-sectional network of PTSD and psychosis in another clinical sample (Hardy et al., 2021). Our findings may instead be particularly relevant to psychological interventions offered in EIP services, from which approximately one-third of participants were recruited.

Our networks should be considered in light of the interval phrasing of ESM items (i.e. ‘Since the last beep…’) used to generate specific PTSD and DSO nodes. A notable advantage of the study, this wording allowed us to better capture the potential infrequency of PTSD symptoms and psychologically reflective nature of DSOs that are likely to be missed by momentary (i.e. ‘Right now…’) wording, as is standard in ESM questionnaire design (Eisele, Kasanova, & Houben, 2022). Strong relationships between items captured within the same timeframe may therefore reflect their temporal grouping (e.g. hallucination nodes being more strongly connected to each other than DSO nodes since they are both momentarily measured) as opposed to specific psychological mechanisms we propose. Additionally, the combination of momentary and interval items obfuscates the interpretation of temporal relationships, for instance, those in the contemporaneous network may reflect relationships in the interval between measurements as opposed to those within a single measurement point. Given this trade-off between measurement against capturing of infrequent but clinically important experiences, it will be important for future work to elucidate whether different timeframes impact network connection estimates. With temporal network analysis in its infancy, it remains unclear whether and how interval items affect outcomes, and we therefore encourage future research to estimate these networks using data across different timeframes to elucidate long-term relationships and psychological mechanisms.

Formal statistical power calculations for network analysis have yet to be devised, though a minimum of 20 observations per participant per node is considered ideal (Epskamp, Borsboom, et al., 2018). As such, our sample size would not have allowed for the reliable estimation of a more complex network including potentially important additional nodes that could facilitate the identification of mechanistic pathways between cPTSD and psychosis. For instance, whether DSOs interact with paranoia *via* appraisals and distress (Garety, Kuipers, Fowler, Freeman, & Bebbington, 2001; Morrison, 2001) or maladaptive coping strategies (e.g. substance use, dissociation; Goldstein et al., 2016; Hyland et al., 2020; Pilton et al., 2015; Swendsen et al., 2011). The exclusive focus on cPTSD in our study provides a foundation for future research that may test such pathways, consistent with the hypothesis-generating nature of network analysis (Epskamp, Maris, Waldorp, & Borsboom, 2018).

As the first study exploring cPTSD and psychosis using complex research and statistical methodologies (i.e. ESM and network analysis) in a clinical population, our study has important theoretical and clinical implications. Our findings indicate that models of PTSD in psychosis apply to people with cPTSD, and provide temporal evidence for multifactorial, reciprocal pathways between post-traumatic sequelae and psychosis (e.g. sense of current threat; disintegrated trauma memories) (Berry, Varese, & Bucci, 2017; Gumley & MacBeth, 2007; Hardy, 2017). The central role of threat is not surprising given the heightened exposure to interpersonal victimization in people with psychosis, and etiological role of threat perception in psychosis (Heriot-Maitland, Wykes, & Peters, 2022; Trauelsen et al., 2015). There is ongoing debate as to the need for stabilization phases in trauma therapies that promote safety and develop helpful emotion regulation strategies (Hoeboer et al., 2021; Ross, Sharma-Patel, Brown, Huntt, & Chaplin, 2021). Insofar as stabilization phases address paranoia and hyperarousal, our findings indicate the importance thereof alongside memory processing in trauma-focused psychosis interventions, as is the case with typical trauma therapies (e.g. trauma-focused cognitive behavioral therapy; eye-movement desensitization and reprocessing therapy [EMDR]) (Hardy et al., 2023; Keen, Hunter, & Peters, 2017; Varese et al., 2023). Ongoing clinical trials of trauma-focused interventions may confirm whether DSOs confer important treatment targets in psychosis (Burger et al., 2022; Karatzias, 2022; Peters et al., 2022). Our findings also indicate that novel interventions aimed specifically at promoting a sense of safety may be especially helpful in people with psychosis and comorbid cPTSD (e.g. Freeman et al., 2024). Indeed, emerging evidence indicates DSOs are responsive to EMDR in early psychosis presentations (Varese et al., 2023).

## Conclusions

Cross-sectional studies have established the mediating role of PTSD in the relationship between traumatic life experiences and psychotic symptoms. Prior network analyses have found specific paths between PTSD and psychosis via trauma-related beliefs and hypervigilance. Our study extends these findings by demonstrating the additional influence of DSOs on psychosis and adds directionality to prior networks that suggest a pivotal role of paranoia in maintaining cPTSD and psychosis. This has important implications for future studies investigating the cPTSD–psychosis comorbidity and the ongoing development of trauma-focused psychosis interventions.

## Supporting information

Panayi et al. supplementary material Panayi et al. supplementary material

## Supplementary material

To view supplementary material for this article, please visit http://doi.org/10.1017/S0033291725000030.
