# Supplementary material for: A temporal network analysis of complex post-traumatic stress disorder and psychosis symptoms
Source: Psychol Med. 2025 Feb 20;55:e43. doi: 10.1017/S0033291725000030 (PMC12055024; doi:10.1017/S0033291725000030)
Supplement: Panayi et al. supplementary material [file S0033291725000030sup001.docx]

**Supplementary Material 1:** R code used to conduct temporal network analysis

#############################################################################

# #

# A temporal network analysis of complex PTSD and psychosis #

# P.Panayi, A. Contreras, et al. #

# Adapted from #

# A. Contreras, C. Valiente, A. Heeren & R. Bentall #

# R code #

# #

#############################################################################

# Load packages:

#---------------

library("mlVAR")

library("graphicalVAR")

library("qgraph")

library(haven)

library(foreign)

library(qgraph)

library(networktools)

library(corpcor)

library(tseries)

library(TSA)

library(tidyverse)

# Please note: The EGA library is not the same as the ega library in CRAN and need to be installed

# using the following commands if it isn't already installed

library("devtools")

devtools::install_github('hfgolino/EGA')

library(IsingFit)

library(networktools)

library(corpcor)

library(EGAnet)

library(mgm)

library(ggplot2)

library(cmprskQR)

library(sessioninfo)

#Packages:

#---------------------------------------------------------------------------------

if (!require(mlVAR)) install.packages("mlVAR"); require(mlVAR)

if (!require(graphicalVAR)) install.packages("graphicalVAR"); require(graphicalVAR)

if (!require(qgraph)) install.packages("qgraph"); require(qgraph)

if (!require(foreign)) install.packages("foreign"); require(foreign)

if (!require(networktools)) install.packages("networktools"); require(networktools)

if (!require(tseries)) install.packages("tseries"); require(tseries)

if (!require(TSA)) install.packages("TSA"); require(TSA)

if (!require(corpcor)) install.packages("corpcor"); require(corpcor)

##################################################################################

# 1.Data preparation #

##################################################################################

#Set working directory and load data:

#------------------------------------

setwd("/Users/peter/Downloads")

dataset <- read_dta("Downloads/Network_analysis_dataset.dta")

#Preparation:

#------------

##Variables are renamed for convenience. The key is as follows:

#Int --> PTSD-intrusions

#Avd --> PTSD-avoidance

#Hpr --> PTSD-hyperarousal

#NSC --> DSO-Negative self-concept

#ED --> DSO-Emotional dysregulation

#ID --> DSO-Interpersonal difficulties

names(dataset) <- c("alias","dayno","beepno","Visions","Voices","Paranoia", "Int", "Avd","Hpr", "NSC","ED", "ID")

vars <- c ("Visions","Voices","Paranoia", "Int", "Avd","Hpr", "NSC","ED", "ID")

idvar <- "alias"

dayvar<-"dayno"

beepvar<-"beepno"

#Assumptions (following Aalbers et al., 2019)

#-------------------------------------------

#Distribution

## Check data distribution

{

pdf("1.Distribution_CPTSD.pdf")

layout(matrix(1:4,2,2))

hist(dataset$Hpr, breaks = 10,xlab = "Observations", main = "PTSD_hyper")

hist(dataset$Avd, breaks = 10,xlab = "Observations", main = "PTSD_avoid")

hist(dataset$Int, breaks = 10,xlab = "Observations", main = "PTSD_mems")

hist(dataset$NSC, breaks = 10, xlab = "Observations", main = "DSO_self")

hist(dataset$ID, breaks = 10, xlab = "Observations", main = "DSO_others")

hist(dataset$ED, breaks = 10, xlab = "Observations", main = "DSO_emotions")

dev.off()

pdf("2.Distribution_PSYCHOSIS.pdf")

layout (matrix(1:4,2,2))

hist(dataset$Paranoia, breaks = 10, xlab = "Observations", main = "Paranoia")

hist(dataset$Voices, breaks = 10, xlab = "Observations", main = "Voices")

hist(dataset$Visions, breaks = 10, xlab = "Observations", main = "Visions")

dev.off()

}

## Normality check Shapiro

{shapiro.test(dataset$Hpr); qqnorm(dataset$Hpr); qqline(dataset$Hpr)

shapiro.test(dataset$Avd); qqnorm(dataset$Avd); qqline(dataset$Avd)

shapiro.test(dataset$Int); qqnorm(dataset$Int); qqline(dataset$Int)

shapiro.test(dataset$NSC); qqnorm(dataset$NSC); qqline(dataset$NSC)

shapiro.test(dataset$ID); qqnorm(dataset$ID); qqline(dataset$ID)

shapiro.test(dataset$ED); qqnorm(dataset$ED); qqline(dataset$ED)

shapiro.test(dataset$Paranoia); qqnorm(dataset$Paranoia); qqline(dataset$Paranoia)

shapiro.test(dataset$Voices); qqnorm(dataset$Voices); qqline(dataset$Voices)

shapiro.test(dataset$Visions); qqnorm(dataset$Visions); qqline(dataset$Visions)}

##Means & SDs

#Calculate the mean (within-person mean) and SD (within-person SD for each participant, on each variable

#Repeated this procedure for all variables in all participants = set of within-person means of all variables.

#From these values, calculated a mean and SD for each variable

#The same, to calculate the within person SD = set of within-person SD for each variable.

#Calculated the mean and SD of these values ("Hpr", "Avd", "Int", "NSC", "ID", "ED", "paranoia", "voices", "visions")

{

# PTSD-hyperarousal

mean_PTSD_hyper <- c()

sd_PTSD_hyper <- c()

for(i in unique(dataset$alias))

{

mean_PTSD_hyper <- c(mean_PTSD_hyper, mean(as.numeric(dataset$Hpr[dataset$alias == i]), na.rm =T))

sd_PTSD_hyper <- c(sd_PTSD_hyper, sd(as.numeric(dataset$Hpr[dataset$alias == i]), na.rm =T))

}

mean(mean_PTSD_hyper)

sd(mean_PTSD_hyper)

t.test(mean_PTSD_hyper)

mean(sd_PTSD_hyper)

sd(sd_PTSD_hyper, na.rm=TRUE)

t.test(sd_PTSD_hyper)

View( t.test(mean_PTSD_hyper))

# PTSD-avoidance

mean_PTSD_avoid <- c()

sd_PTSD_avoid <- c()

for(i in unique(dataset$alias))

{

mean_PTSD_avoid <- c(mean_PTSD_avoid, mean(as.numeric(dataset$Avd[dataset$alias == i]), na.rm =T))

sd_PTSD_avoid <- c(sd_PTSD_avoid, sd(as.numeric(dataset$Avd[dataset$alias == i]), na.rm =T))

}

mean(mean_PTSD_avoid)

sd(mean_PTSD_avoid)

t.test(mean_PTSD_avoid)

mean(sd_PTSD_avoid)

sd(sd_PTSD_avoid, na.rm=TRUE)

t.test(sd_PTSD_avoid)

# PTSD-intrusive-memories

mean_PTSD_mems <- c()

sd_PTSD_mems <- c()

for(i in unique(dataset$alias))

{

mean_PTSD_mems <- c(mean_PTSD_mems, mean(as.numeric(dataset$Int[dataset$alias == i]), na.rm =T))

sd_PTSD_mems <- c(sd_PTSD_mems, sd(as.numeric(dataset$Int[dataset$alias == i]), na.rm =T))

}

mean(mean_PTSD_mems)

sd(mean_PTSD_mems)

t.test(mean_PTSD_mems)

mean(sd_PTSD_mems)

sd(sd_PTSD_mems, na.rm=TRUE)

t.test(sd_PTSD_mems)

# DSO-self

mean_DSO_self <- c()

sd_DSO_self <- c()

for(i in unique(dataset$alias))

{

mean_DSO_self <- c(mean_DSO_self, mean(as.numeric(dataset$NSC[dataset$alias == i]), na.rm =T))

sd_DSO_self <- c(sd_DSO_self, sd(as.numeric(dataset$NSC[dataset$alias == i]), na.rm =T))

}

mean(mean_DSO_self)

sd(mean_DSO_self)

t.test(mean_DSO_self)

mean(sd_DSO_self)

sd(sd_DSO_self)

t.test(sd_DSO_self)

# DSO-others

mean_DSO_other <- c()

sd_DSO_other <- c()

for(i in unique(dataset$alias))

{

mean_DSO_other <- c(mean_DSO_other, mean(as.numeric(dataset$ID[dataset$alias == i]), na.rm =T))

sd_DSO_other <- c(sd_DSO_other, sd(as.numeric(dataset$ID[dataset$alias == i]), na.rm =T))

}

mean(mean_DSO_other)

sd(mean_DSO_other)

t.test(mean_DSO_other)

mean(sd_DSO_other)

sd(sd_DSO_other, na.rm=TRUE)

t.test(sd_DSO_other)

# DSO-emotions

mean_DSO_emotions <- c()

sd_DSO_emotions <- c()

for(i in unique(dataset$alias))

{

mean_DSO_emotions <- c(mean_DSO_emotions, mean(as.numeric(dataset$ED[dataset$alias == i]), na.rm =T))

sd_DSO_emotions <- c(sd_DSO_emotions, sd(as.numeric(dataset$ED[dataset$alias == i]), na.rm =T))

}

mean(mean_DSO_emotions)

sd(mean_DSO_emotions)

t.test(mean_DSO_emotions)

mean(sd_DSO_emotions)

sd(sd_DSO_emotions, na.rm=TRUE)

t.test(sd_DSO_emotions)

# Paranoia

mean_paranoia <- c()

sd_paranoia <- c()

for(i in unique(dataset$alias))

{

mean_paranoia <- c(mean_paranoia, mean(as.numeric(dataset$Paranoia[dataset$alias== i]), na.rm =T))

sd_paranoia <- c(sd_paranoia, sd(as.numeric(dataset$Paranoia[dataset$alias == i]), na.rm =T))

}

t.test(sd_paranoia)

mean(mean_paranoia)

sd(mean_paranoia)

t.test(mean_paranoia)

mean(sd_paranoia)

sd(sd_paranoia, na.rm=TRUE)

t.test(sd_paranoia)

# Voices

mean_voices <- c()

sd_voices <- c()

for(i in unique(dataset$alias))

{

mean_voices <- c(mean_voices, mean(as.numeric(dataset$Voices[dataset$alias== i]), na.rm =T))

sd_voices <- c(sd_voices, sd(as.numeric(dataset$Voices[dataset$alias == i]), na.rm =T))

}

t.test(sd_voices)

mean(mean_voices)

sd(mean_voices)

t.test(mean_voices)

mean(sd_voices)

sd(sd_voices)

t.test(sd_voices)

# Visions

mean_visions <- c()

sd_visions <- c()

for(i in unique(dataset$alias))

{

mean_visions <- c(mean_visions, mean(as.numeric(dataset$Visions[dataset$alias== i]), na.rm =T))

sd_visions <- c(sd_visions, sd(as.numeric(dataset$Visions[dataset$alias == i]), na.rm =T))

}

t.test(sd_visions)

mean(mean_visions)

sd(mean_visions)

t.test(mean_visions)

mean(sd_visions)

sd(sd_visions)

t.test(sd_visions)

##Normality check for mean levels

{

mean_levels <- cbind(mean_PTSD_hyper,

mean_PTSD_avoid,

mean_PTSD_mems,

mean_DSO_emotions,

mean_DSO_other,

mean_DSO_self,

mean_paranoia,

mean_voices,

mean_visions)

for(i in 1:9)

{

print(colnames(mean_levels)[i])

print(shapiro.test(mean_levels[,i]))

}

}

#Stationarity. Kwiatkowksi-Phillips-Schmidt-Shin unit root test (following Bringmann, 2016; Kwiatkowski,Phillips, Schmidt, & Shin, 1992).

#Check if variables change as a function of parameters (level) or time (trend)

#It returns a list class "htest" containing the following components:

# a) statistic: the value of the test statistic

# b) parameter: the truncations lag parameter

# c) p.value: the p-value of the test

# d) method: a character string indiciating what type of test was performed

# e) data.name: a character string giving the name of the data

#KPSS Test for Level Stationarity

# H0: level stationarity

# H1: unit root [not level stationary]

{

kpps_p <- c()

for(i in unique(dataset$alias))

{

randomness <- tseries::kpss.test(na.exclude(dataset$Hpr[dataset$alias == i]), lshort = TRUE, null = "Level")

kpps_p <- c(kpps_p, randomness$p.value)

}

kpps_p <- cbind(unique(dataset$alias),kpps_p )

kpps_p [,2] < 0.05/125

kpps_p <- c()

for(i in unique(dataset$alias))

{

randomness <- tseries::kpss.test(na.exclude(dataset$Avd[dataset$alias == i]), lshort = TRUE, null = "Level")

kpps_p <- c(kpps_p, randomness$p.value)

}

kpps_p <- cbind(unique(dataset$alias),kpps_p )

kpps_p [,2] < 0.05/125

kpps_p <- c()

for(i in unique(dataset$alias))

{

randomness <- tseries::kpss.test(na.exclude(dataset$Int[dataset$alias == i]), lshort = TRUE, null = "Level")

kpps_p <- c(kpps_p, randomness$p.value)

}

kpps_p <- cbind(unique(dataset$alias),kpps_p )

kpps_p [,2] < 0.05/125

kpps_p <- c()

for(i in unique(dataset$alias))

{

randomness <- tseries::kpss.test(na.exclude(dataset$ED[dataset$alias == i]), lshort = TRUE, null = "Level")

kpps_p <- c(kpps_p, randomness$p.value)

}

kpps_p <- cbind(unique(dataset$alias),kpps_p )

kpps_p [,2] < 0.05/125

##This returns a significant result. As such, the ADF test is used to confirm this:

adf.test(na.exclude(dataset$ED, alternative = "explosive", k = 0)) ##Suggests it is stationary

kpps_p <- c()

for(i in unique(dataset$alias))

{

randomness <- tseries::kpss.test(na.exclude(dataset$ID[dataset$alias == i]), lshort = TRUE, null = "Level")

kpps_p <- c(kpps_p, randomness$p.value)

}

kpps_p <- cbind(unique(dataset$alias),kpps_p )

kpps_p [,2] < 0.05/125

kpps_p <- c()

for(i in unique(dataset$alias))

{

randomness <- tseries::kpss.test(na.exclude(dataset$NSC[dataset$alias == i]), lshort = TRUE, null = "Level")

kpps_p <- c(kpps_p, randomness$p.value)

}

kpps_p <- cbind(unique(dataset$alias),kpps_p )

kpps_p [,2] < 0.05/125

kpps_p <- c()

for(i in unique(dataset$alias))

{

randomness <- tseries::kpss.test(na.exclude(dataset$Paranoia[dataset$alias == i]), lshort = TRUE, null = "Level")

kpps_p <- c(kpps_p, randomness$p.value)

}

kpps_p <- cbind(unique(dataset$alias),kpps_p )

kpps_p [,2] < 0.05/125

kpps_p <- c()

for(i in unique(dataset$alias))

{

randomness <- tseries::kpss.test(na.exclude(dataset$Visions[dataset$alias == i]), lshort = TRUE, null = "Level")

kpps_p <- c(kpps_p, randomness$p.value)

}

kpps_p <- cbind(unique(dataset$alias),kpps_p )

kpps_p [,2] < 0.05/125

kpps_p <- c()

for(i in unique(dataset$alias))

{

randomness <- tseries::kpss.test(na.exclude(dataset$Voices[dataset$alias == i]), lshort = TRUE, null = "Level")

kpps_p <- c(kpps_p, randomness$p.value)

}

kpps_p <- cbind(unique(dataset$alias),kpps_p )

kpps_p [,2] < 0.05/125

}

View(randomness)

#KPSS Test for Trend Stationarity

# H0: trend stationarity

# H1: not trend stationary

{

kpps_p <- c()

for(i in unique(dataset$alias))

{

randomness <- tseries::kpss.test(na.exclude(dataset$Hpr[dataset$alias == i]), lshort = TRUE, null = "Trend")

kpps_p <- c(kpps_p, randomness$p.value)

}

kpps_p <- cbind(unique(dataset$alias),kpps_p )

kpps_p [,2] < 0.05/125

kpps_p <- c()

for(i in unique(dataset$alias))

{

randomness <- tseries::kpss.test(na.exclude(dataset$Avd[dataset$alias == i]), lshort = TRUE, null = "Trend")

kpps_p <- c(kpps_p, randomness$p.value)

}

kpps_p <- cbind(unique(dataset$alias),kpps_p )

kpps_p [,2] < 0.05/125

kpps_p <- c()

for(i in unique(dataset$alias))

{

randomness <- tseries::kpss.test(na.exclude(dataset$Int[dataset$alias == i]), lshort = TRUE, null = "Trend")

kpps_p <- c(kpps_p, randomness$p.value)

}

kpps_p <- cbind(unique(dataset$alias),kpps_p )

kpps_p [,2] < 0.05/125

kpps_p <- c()

for(i in unique(dataset$alias))

{

randomness <- tseries::kpss.test(na.exclude(dataset$ED[dataset$alias == i]), lshort = TRUE, null = "Trend")

kpps_p <- c(kpps_p, randomness$p.value)

}

kpps_p <- cbind(unique(dataset$alias),kpps_p )

kpps_p [,2] < 0.05/125

##This returns a significant result. As such, the ADF test is used to confirm this:

adf.test(na.exclude(dataset$ED, alternative = "stationary", k = 0)) ##Suggests it is stationary

kpps_p <- c()

for(i in unique(dataset$alias))

{

randomness <- tseries::kpss.test(na.exclude(dataset$ID[dataset$alias == i]), lshort = TRUE, null = "Trend")

kpps_p <- c(kpps_p, randomness$p.value)

}

kpps_p <- cbind(unique(dataset$alias),kpps_p )

kpps_p [,2] < 0.05/125

kpps_p <- c()

for(i in unique(dataset$alias))

{

randomness <- tseries::kpss.test(na.exclude(dataset$NSC[dataset$alias == i]), lshort = TRUE, null = "Trend")

kpps_p <- c(kpps_p, randomness$p.value)

}

kpps_p <- cbind(unique(dataset$alias),kpps_p )

kpps_p [,2] < 0.05/125

##This returns a significant result. As such, the ADF test is used to confirm this:

adf.test(na.exclude(dataset$NSC, alternative = "stationary", k = 0)) ##Suggests it is stationary

kpps_p <- c()

for(i in unique(dataset$alias))

{

randomness <- tseries::kpss.test(na.exclude(dataset$Paranoia[dataset$alias == i]), lshort = TRUE, null = "Trend")

kpps_p <- c(kpps_p, randomness$p.value)

}

kpps_p <- cbind(unique(dataset$alias),kpps_p )

kpps_p [,2] < 0.05/125

kpps_p <- c()

for(i in unique(dataset$alias))

{

randomness <- tseries::kpss.test(na.exclude(dataset$Visions[dataset$alias == i]), lshort = TRUE, null = "Trend")

kpps_p <- c(kpps_p, randomness$p.value)

}

kpps_p <- cbind(unique(dataset$alias),kpps_p )

kpps_p [,2] < 0.05/125

kpps_p <- c()

for(i in unique(dataset$alias))

{

randomness <- tseries::kpss.test(na.exclude(dataset$Voices[dataset$alias == i]), lshort = TRUE, null = "Trend")

kpps_p <- c(kpps_p, randomness$p.value)

}

kpps_p <- cbind(unique(dataset$alias),kpps_p )

kpps_p [,2] < 0.05/125

}

################################################################################################

# 2. Time-series n>1 analysis #

################################################################################################

#Estimation: two-step multilevel Vector Autoregressive (mlVAR):

#--------------------------------------------------------------

mlVAR <- mlVAR(dataset, vars, idvar, lags = 1, estimator = "lmer",

temporal = "correlated", contemporaneous = "correlated")

#Visualization:

#--------------

#Separated plot:

contemporaneous<-plot(mlVAR, "contemporaneous", layout = "circle", nonsig = "hide", theme = "colorblind", title = "Contemporaneous",

labels = vars, vsize = 13, rule = "and", asize = 10, mar = c(5,5,5,5))

temporal<-plot(mlVAR, "temporal", layout = "circle", nonsig = "hide", theme = "colorblind", title = "Temporal",

labels = vars, vsize = 13, rule = "and", asize = 5, mar = c(5,5,5,5))

between_subjects<-plot(mlVAR, "between", layout = "circle", nonsig = "hide", theme = "colorblind", title = "Between-subjects",

labels = vars, vsize = 13, rule = "and", asize = 10, mar = c(5,5,5,5))

#To plot the networks with symptom clusters separated by colour

#names of the symptoms

symptoms<-c("visions", "voices", "paranoia", "Int", "Avd",

"Hpr", "NSC", "ED", "ID")

# group defined here

gr<- list(c(1:3), c(4:6), c(7:9)) #psychosis, PTSD & DSO symptom clusters

# Clusters defined here

Clusters <- c(rep("psychosis",3), rep("PTSD", 3), rep("DSO", 3))

#Plot contemporaneous network:

pdf("contemporaneous_network.pdf")

plot(mlVAR, "contemporaneous", layout = "circle", groups=Clusters, nonsig = "hide", theme = "colorblind", title = "Contemporaneous",

labels = vars, vsize = 13, rule = "and", asize = 10, mar = c(5,5,5,5))

dev.off()

#Plot temporal network:

pdf("temporal_network.pdf",width=10,height=9)

plot(mlVAR, "temporal", layout = "circle", groups=Clusters, nonsig = "hide", theme = "colorblind", title = "Temporal",

labels = vars, vsize = 10, rule = "and", asize = 5, mar = c(5,5,5,5))

dev.off()

#Plot between network:

pdf("between_network.pdf")

plot(mlVAR, "between", layout = "circle", groups=Clusters, nonsig = "hide", theme = "colorblind", title = "Between-subjects",

labels = vars, vsize = 13, rule = "and", asize = 10, mar = c(5,5,5,5))

dev.off()

##summary of model

summary(mlVAR, show=c("fit", "temporal", "contemporaneous"), round=3)

temporal

contemporaneous

between_subjects

##centrality plot

centralityTable(temporal)

pdf("centrality.pdf")

centralityPlot(temporal, signed=FALSE, labels =symptoms)

dev.off()

##Clustering metrics

#Extract matrix from between subjects network

matrix<- getWmat(between_subjects)

#Run EGA to generate clusters

Ega <- EGA.fit(data = matrix, model = "glasso", uni.method = "LE", plot.EGA = TRUE, n=155, singleton=FALSE)

print(Ega)

pdf("clustering.pdf")

plot(Ega)

dev.off()

#Check package versions, etc.

session_info()

**Supplementary Table 1.**

*Shapiro-Wilk normality test values.*

| **Variable** | **Statistic** | ***p*** |
| --- | --- | --- |
| Memory intrusions | .98384 | .067 |
| Avoidance | .97975 | .022 |
| Hyperarousal | .98648 | .137 |
| Emotional dysregulation | .98799 | .205 |
| Interpersonal difficulties | .9815 | .036 |
| Negative self-concept | .98607 | .122 |
| Paranoia | .98244 | .046 |
| Voices | .90198 | <.001 |
| Visions | .88097 | <.001 |

*^Note:^* ^p^ *^< .05 indicates a non-normal distribution^*

**Supplementary Table 2.**

*KPSS unit root test values*

| **Variable** | **Level Statistic** | **Trend Statistic** |
| --- | --- | --- |
| Memory intrusions | .19970 | .07220 |
| Avoidance | .08818 | .06804 |
| Hyperarousal | .14623 | .08984 |
| Emotional dysregulation | .51179^1^ | .15328^2^ |
| Interpersonal difficulties | .08559 | .08279 |
| Negative self-concept | .27379 | .15612^3^ |
| Paranoia | .33918 | .06561 |
| Voices | .09030 | .06307 |
| Visions | .09454 | .07189 |

*^Note:^* ^p^ *^< .05 indicates non-stationarity; all^* ^p^*^’s = .1, except 1^*^p^ *^= .039, 2^*^p^ *^= .044, 3^*^p^ *^= .042^*

**Supplementary Table 3.** Partial correlation coefficients of between-subject network edges.

|  | **Voices** | **Visions** | **Paranoia** | **Intrusions** | **Avoidance** | **Hyperarousal** | **Emotional dysregulation** | **Negative self-concept** | **Interpersonal difficulties** |
| --- | --- | --- | --- | --- | --- | --- | --- | --- | --- |
| **Voices** | - | .475 | .004 | .057 | -.035 | .219 | .173 | -.047 | -.122 |
| **Visions** |  | - | .112 | .182 | -.007 | -.068 | .159 | .036 | -.105 |
| **Paranoia** |  |  | - | .279 | .091 | .227 | -.029 | .116 | .197 |
| **Intrusions** |  |  |  | - | .369 | .046 | .205 | .039 | .082 |
| **Avoidance** |  |  |  |  | - | .207 | .111 | -.145 | -.064 |
| **Hyperarousal** |  |  |  |  |  | - | .135 | .075 | .158 |
| **Emotional dysregulation** |  |  |  |  |  |  | - | .225 | .415 |
| **Negative self-concept** |  |  |  |  |  |  |  | - | .147 |
| **Interpersonal difficulties** |  |  |  |  |  |  |  |  | - |

*Note*: all *p*’s => .05

**Supplementary Table 4.** Partial correlation coefficients of contemporaneous network edges.

|  | **Voices** | **Visions** | **Paranoia** | **Intrusions** | **Avoidance** | **Hyperarousal** | **Emotional dysregulation** | **Negative self-concept** | **Interpersonal difficulties** |
| --- | --- | --- | --- | --- | --- | --- | --- | --- | --- |
| **Voices** | - | .325 | .160 | .054 | .023 | .043 | .055 | .067 | .029 |
| **Visions** |  | - | .076 | .051 | .019 | .015 | .027 | .051 | -.007 |
| **Paranoia** |  |  | - | .033 | .018 | .184 | .087 | .180 | .096 |
| **Intrusions** |  |  |  | - | .140 | .158 | .162 | .089 | -.012 |
| **Avoidance** |  |  |  |  | - | .106 | .066 | .030 | .069 |
| **Hyperarousal** |  |  |  |  |  | - | .189 | .046 | .070 |
| **Emotional dysregulation** |  |  |  |  |  |  | - | .130 | .214 |
| **Negative self-concept** |  |  |  |  |  |  |  | - | -.005 |
| **Interpersonal difficulties** |  |  |  |  |  |  |  |  | - |

*Note*: all *p*’s => .05

**Supplementary Table 5.** Regression coefficients of temporal network edges. Direction of relationships begin from variables in column 1 predicting those in row 1 (such that the leading diagonal represents autoregressive loops).

|  | **Voices** | **Visions** | **Paranoia** | **Intrusions** | **Avoidance** | **Hyperarousal** | **Emotional dysregulation** | **Negative self-concept** | **Interpersonal difficulties** |
| --- | --- | --- | --- | --- | --- | --- | --- | --- | --- |
| **Voices** | .108*** | .046 | -.007 | .048 | .023 | -.030 | .007 | -.012 | -.031 |
| **Visions** | .059 | .107*** | .048 | .007 | .033 | .059 | .058 | .061* | .046 |
| **Paranoia** | .044* | .014 | .169*** | .033 | .061* | .096*** | .058* | .044* | .039 |
| **Intrusions** | .004 | .014 | .009 | .123*** | .008 | .019 | .021 | -.003 | .049* |
| **Avoidance** | .023 | -.014 | .003 | -.017 | .131*** | -.015 | -.032 | .025 | .024 |
| **Hyperarousal** | .012 | .006 | .046* | .018 | .006 | .181*** | .014 | .024 | .006 |
| **Emotional dysregulation** | .018 | -.007 | .057* | .068* | -.007 | .030 | .177*** | .043 | .067* |
| **Negative self-concept** | .025 | .003 | .047* | .031 | .003 | -.008 | .029 | .220*** | .038 |
| **Interpersonal difficulties** | .015 | .032 | .029 | .017 | .059** | .048* | .056** | .025 | .117*** |

*Note*: **p < .05; **p < .01; ***p < .001*

**Supplementary Figure 1.**

*Distributions of ESM cPTSD scores*


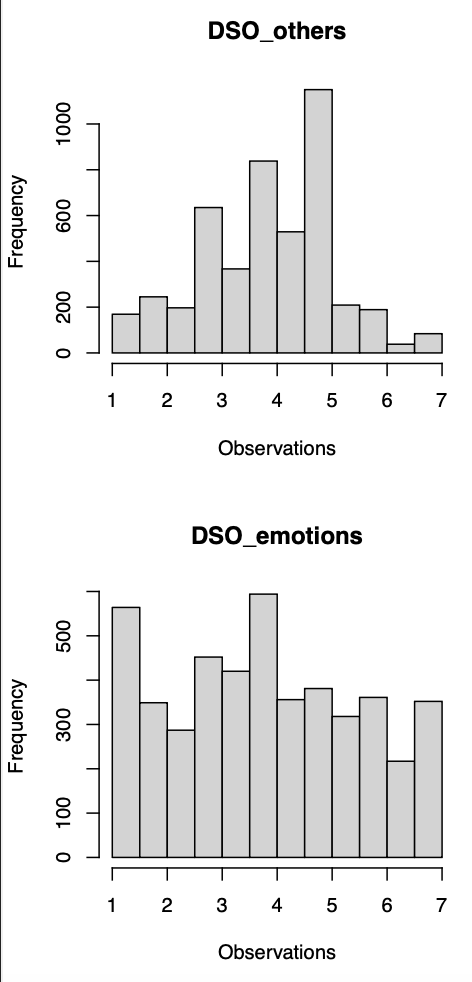

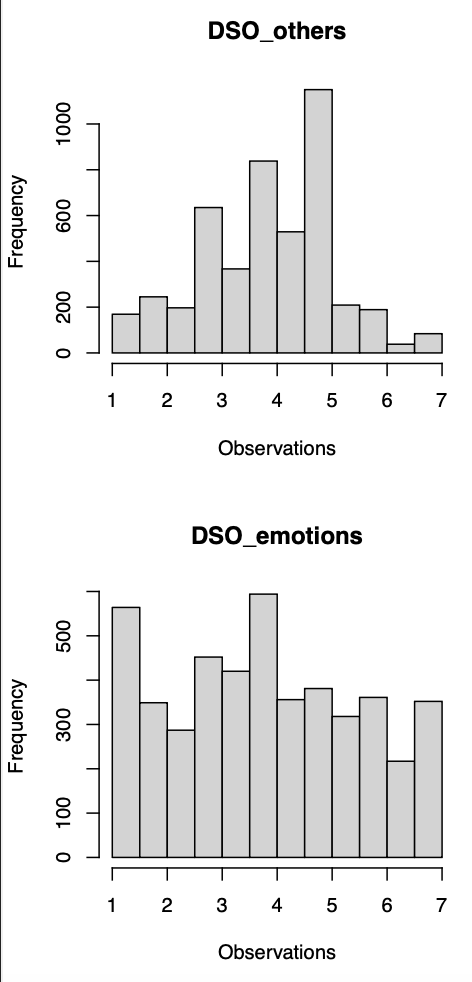


**Supplementary Figure 2.**

*Distributions of ESM psychosis scores*

**Supplementary Figure 3.**

*Centrality plots*

*^Note: Hpr = Hyperarousal, Int = Intrusions, Avd = Avoidance, ID = Interpersonal Difficulties, ED = Emotional Dysregulation, NSC = Negative Self-Concept^*

**Supplementary Figure 4.**

*Clustering analysis
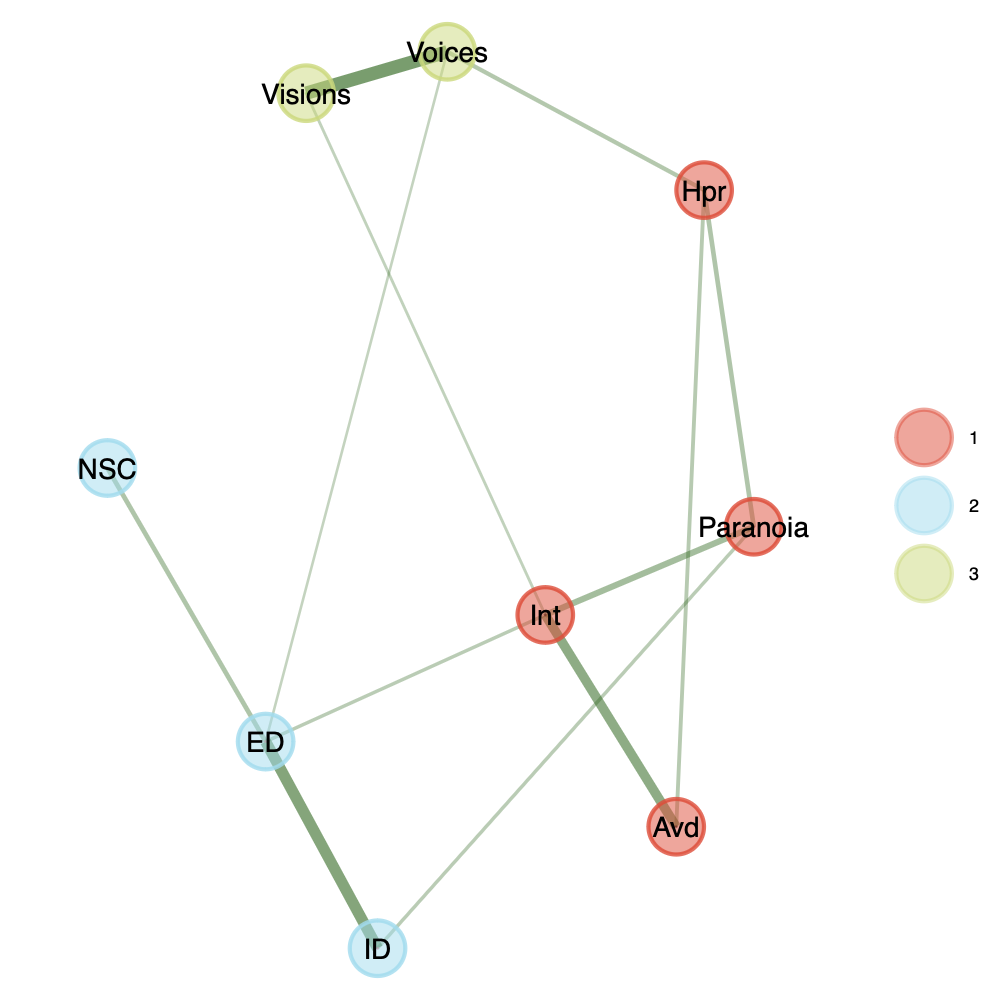
*

*^Note: Hpr = Hyperarousal, Int = Intrusions, Avd = Avoidance, ID = Interpersonal Difficulties, ED = Emotional Dysregulation, NSC = Negative Self-Concept. Colours refer to clusters identified in the analysis^*
